# Diversity of Virulence Phenotypes among Type III Secretion Negative *Pseudomonas aeruginosa* Clinical Isolates

**DOI:** 10.1371/journal.pone.0086829

**Published:** 2014-01-23

**Authors:** Jonida Toska, Yan Sun, Dalina Alvarez Carbonell, Altreisha N. -S. Foster, Michael R. Jacobs, Eric Pearlman, Arne Rietsch

**Affiliations:** 1 Department of Molecular Biology and Microbiology, Case Western Reserve University, Cleveland, Ohio, United States of America; 2 Department of Ophthalmology and Visual Sciences, Case Western Reserve University, Cleveland, Ohio, United States of America; 3 Department of Pathology, Case Western Reserve University and University Hospitals Case Medical Center, Cleveland, Ohio, United States of America; UC Berkeley, United States of America

## Abstract

*Pseudomonas aeruginosa* is a frequent cause of acute infections. The primary virulence factor that has been linked to clinical disease is the type III secretion system, a molecular syringe that delivers effector proteins directly into host cells. Despite the importance of type III secretion in dictating clinical outcomes and promoting disease in animal models of infections, clinical isolates often do not express the type III secretion system *in vitro*. Here we screened 81 clinical *P. aeruginosa* isolates for secretion of type III secretion system substrates by western blot. Non-expressing strains were also subjected to a functional test assaying the ability to intoxicate epithelial cells *in vitro*, and to survive and cause disease in a murine model of corneal infection. 26 of 81 clinical isolates were found to be type III secretion negative by western blot. 17 of these 26 non-expressing strains were tested for their ability to cause epithelial cell rounding. Of these, three isolates caused epithelial cell rounding in a type III secretion system dependent manner, and one strain was cytotoxic in a T3SS-independent manner. Five T3SS-negative isolates were also tested for their ability to cause disease in a murine model of corneal infection. Of these isolates, two strains caused severe corneal disease in a T3SS-independent manner. Interestingly, one of these strains caused significant disease (inflammation) despite being cleared. Our data therefore show that *P. aeruginosa* clinical isolates can cause disease in a T3SS-independent manner, demonstrating the existence of novel modifiers of clinical disease.

## Introduction

*Pseudomonas aeruginosa* is a common nosocomial pathogen responsible for acute lung infections, blood stream and catheter-associated infections [1]–[3]. It is also a common cause of corneal infections due to contaminated contact lenses or associated with agricultural work [4]–[8]. One of the most important virulence mechanisms is the type III secretion system, which directly injects effector proteins into targeted host cells [9]. Type III secretion also allows *P. aeruginosa* to evade killing by infiltrating neutrophils [10], [11].

Evasion of killing by neutrophils is a function of the effector proteins injected by *P. aeruginosa*. To date, four effector proteins have been described in *P. aeruginosa*: exoenzyme S (ExoS), ExoT, ExoU and ExoY. ExoS and ExoT are heterobifunctional enzymes with an amino-terminal Rho-GAP domain and a C-terminal ADP ribosyltransferase domain [12]. ExoU is a potent phospholipase [13] and ExoY is an adenylate cyclase [14]. The primary effectors associated with survival in mammalian models of infection are ExoS, ExoT and ExoU [15]–[20]. The presence or absence of ExoY generally does not seem to impact virulence greatly [11], [19], [20]. Analysis of the effector complement produced by strains of *P. aeruginosa* demonstrated that most isolates produce ExoT. Distribution of ExoS and ExoU, on the other hand, is generally mutually exclusive [1], [2], [21], [22].

Detectable secretion of type III effectors *in vitro* correlates with increased morbidity, higher rates of mortality and relapse in *P. aeruginosa* ventilator-associated pneumonia and blood stream infections [2], [23], [24]. Despite the prominent role that type III secretion plays in animal models of infection, and its link to poor outcomes in the clinic, many clinical isolates of *P. aeruginosa* are T3SS-negative when assayed *in vitro* [2], [23]–[26]. This observation raises the question if the apparent lack of T3SS gene expression is due to a regulatory difference in these strains, which allows expression *in vivo*, but not in culture *in vitro*. Alternatively, the bacteria could have been passengers that colonized an infected host without being the primary pathogen associated with disease, or, they could be expressing other virulence factors. One report showed that eight clinical isolates from acute P. aeruginosa infections were type III secretion negative isolates, and that three displayed moderate virulence in an animal model of acute lung infection and one other strain retained a limited amount of cytotoxicity against epithelial cells. It was not determined if the observed virulence phenotypes were due to a poorly expressed type III secretion system, or due to other known or novel virulence factors [2].

Here we characterized 81 clinical isolates from patients with infections of the urinary tract, blood stream, wound infections and acute infections of the respiratory tract. We found that 26 were T3SS negative by western blot. Since all except one of the 26 strains still had type III secretion genes on the chromosome, we generated type III secretion null mutants in 16 strains and determined if they cause cytotoxicity *in vitro.* We also examined five T3SS-negative strains and their defined T3SS- mutant derivatives for virulence in an animal model of infection. Taken together, our data indicate that strains that do not express detectable levels of T3SS effector and translocator proteins comprise a complex set of phenotypic groups, including strains that can persist and cause disease in a T3SS-independent manner.

## Materials and Methods

### Ethics Statement

Clinical isolates were de-identified with only the source of the infection passed on to the investigators. The experiments were classified as non-human subject research under 45 CFR46/21 CFR 56 and HIPAA exempt by the University Hospitals Case Medical Center IRB.

All animals were housed under specific pathogen-free conditions in microisolator cages and maintained according to institutional guidelines and the Association for Research in Vision and Ophthalmology Statement for the Use of Animals in Ophthalmic and Vision Research. The infection protocol was approved by the University Hospitals Case Medical Center IACUC [protocol #2012–0105 (E.P.)].

### Bacterial Strains and Plasmids and PCR Primers

*P. aeruginosa* and *E. coli* strains were routinely cultured on Luria-Bertani medium (LB). *E. coli* strains harboring plasmids encoding a gentamicin resistance gene were cultured on LB plates with 15 µg/mL gentamicin, buffered with 10 mM NaPO4 (pH6.9). *P. aeruginosa* strains with an integrated plasmid conferring gentamicin resistance were cultured on LB plates with 30 µg/mL gentamicin and 5 µg/mL triclosan (Sigma). *E. coli* strain DH5α was used for plasmid construction and strain SM10 λ*pir* [27] was used to transfer plasmids into *P. aeruginosa* by conjugation. *P. aeruginosa* strains PAO1F and 19660 have been described previously [28], [29]. Clinical isolates were obtained from the microbiology lab in the Pathology department at University Hospitals Case Medical Center.

Plasmids pEXG2-Δ*popD* and pEXG2-Δ*pscD* have been described previously [11], [30]. Plasmid pINT-*pscC1* was constructed by amplifying an internal fragment of *pscC* (codons 20–417) using primers intC1-5R (5′-AAAAAGAATTCGCGGACTGCTGGCGCTGCTGCCTG-3′) and intC1-3H (5′-AAAAAAAGCTTGGAAGGGTCGGCACGCCAGCCGAAG-3′) and cloning them as an *Eco*RI/*Hin*dIII fragment into plasmid pINT [31]. Plasmid pEXG2Δ*pscUL* was constructed by amplifying flanking regions 3′ of genes *pscU* and *pscL* using primers *pscU*-3-1 (5′-AAAAAaagcttGCCCGTGGCAACGAGGAACTGGACAA-3′) and *pscU*-3-2 (5′-TTCAGCATGCTTGCGGCTCGAGTTACGCCCTGATCGCTATCGGGCAGGGCA-3′) and *pscL*-3-1 (5′-AACTCGAGCCGCAAGCATGCTGAAGGGGACGCCGGTTGAGGGAACCA-3′) and *pscL*-3-2 (5′-AAAAAtctagaAGGCGATGCTCGAGGAGGCCTT-3′), respectively. The two flanking regions were joined by splicing-by-overlap extension PCR (SOE-PCR, [32]) and cloned into plasmid pEXG2 as an *Eco*RI/*Hin*dIII fragment.

Null mutations of type III secretion genes were constructed by allelic exchange (Δ*pscD,* Δ*popD* and Δ*pscUL*) or by insertion of a non-replicating plasmid (*pscC-*). The plasmids were transferred to *P. aeruginosa* by conjugation using *E. coli* strain SM10 λ*pir*, where they integrated into the targeted locus by homologous recombination. Cointegrates were selected on plates with 30 µg/ml gentamicin and 5 µg/mL triclosan (to select against the *E. coli* donor). In the case of the allelic exchange procedure, strains in which the plasmid had exited *via* a second homologous recombination event were isolated using 5% sucrose plates as a counterselection (10 g/L tryptone, 5 g/L yeast extract, 50 g/L sucrose and 15 g/L agar), since plasmid pEXG2 harbors a *sacB* gene that confers sensitivity to sucrose in the medium [31]. Presence of the deletion was confirmed by PCR.

Presence of *exoS*, *exoT*, *exoU*, *pscU*, *pscL*, as well as the absence of the T3SS apparatus genes were tested by PCR using the following primer sets: *exoS* [ORF1-5-1 (5′-TTCAGCATGCTTGCGGCTCGAGTTATTCATGGCGTGTTCCGAGTCA-3′) and ORF1-5-2 (5′-AAAAAGAATTCGCCCAGCCAGTCCATGATCGCCA-3′)], *exoU* [exoU-3H (5′-AAAAAAAGCTTTCATGTGAACTCCTTATTCCGCCAAG-3′) and exoUD344A-3-1 (5′-AGTAGAGTGGACAGAATTCCAGGcaGGCGGGGTGATGATTAACGTG-3′)], *exoT* [exoT3-2 (5′-AAAAAAAAGCTTCTAGCCGACCCGTGTGCGGAAAAGA-3′) and exoTE383/385D-3-1 (5′-ATCGATCGAGGGCGATGATCAGGATATCCTCTACGACAAG-3′)], *pscU* [pscU5X (5′-AAAAAATCTAGAGGAGGAGACGCCATGAGCGCCGAGAAGA-3′) and pscU3H (5′-AAAAAAAAGCTTGATAGCGATCAGGGCGTATCCGTCTGCT-3′)], *pscL* [pscL5R (5′-AAAAAAGAATTCGGAGGGCGATGAATGCTTCCATTTGTT-3′) and pscL3H (5′-AAAAAAAAGCTTTCAACCGGCGTCCCCTTCCTCCT-3′)] and absence of the T3SS genes [PA1689to3 (5′-ATCGTTCATCCAGACCGCGACCAAGAG-3′) and bglXto5 (5′-AGCAGGACCCGGTCGCCAGCCTCAG-3′)].

PCR was performed with Denville Taq Blue using the manufacturer’s buffer but with the addition of DMSO to 5%.

### Protein Secretion Profiles

For secretion assays *P. aeruginosa* strains were grown overnight in a modified LB-medium with 200 mM NaCl, 10 mM MgCl_2_ and 0.5 mM CaCl_2_ (LB-MC). On the day of the experiment bacteria were diluted 1∶300 into 3 mL LB-MC from which calcium had been removed by adding EGTA to 5 mM (final concentration) and grown to mid-logarithmic phase. 1 mL of each culture was removed into a microcentrifuge tube. The bacteria were pelleted by centrifugation and 0.5 mL of the supernatant was transferred into a second microcentrifuge tube. Supernatant proteins were precipitated by adding trichloroacetic acid to a final concentration of 10% and pelleted by centrifugation. Protein pellets were washed 1x with acetone, dried and resuspended in 1x SDS sample buffer normalized for OD_600_. Samples were separated by SDS-PAGE and either stained for total protein using SYPRO Ruby (Invitrogen) according to the manufacturer’s instructions or transferred to PVDF membrane and probed for the presence of ExoS, ExoT, ExoU, PopB and PopD [30] by western blot. Primary antibodies were detected with a HRP-conjugated anti-rabbit secondary antibody (Sigma) and WesternBright Quantum detection reagent (Advansta) using a GE ImageQuant LAS 4000 digital imaging system.

### Affinity Purification of Antibodies

Antibodies against ExoS, ExoT, PopB and PopD were purified following a published protocol [33]. The antigens used for the purification consisted of ExoS(aa 74–453), ExoT(aa 74–457), full-length PopB or PopD lacking the transmembrane region (PopDΔTM, lacking aa 108–146), respectively. Briefly, the antigen was resuspended at a concentration of 1 mg/mL in 20 mM HEPES pH 7.6,100 mM NaCl, 10% glycerol and coupled to Affigel-10 resin according to the manufacturer’s instructions (BioRad). After coupling of the antigen, the resin was washed with 10 mL PBS, 10 mL PBS with 500 mM NaCl, 0.1% Tween-20, 10 mL 0.2x PBS, 10 mL 100 mM glycine (pH2.5) and 20 mL PBS at which point the resin was transferred into a conical tube and rotated together with 5–10 mL of crude antisera overnight at 4°C. The bead slurry was then transferred to a column, the serum was passed through the column and passed over the column a second time. The column was then washed 10 mL PBS, 40 mL PBS with 500 mM NaCl, 0.1% Tween-20 and 10 mL 0.2x PBS. The bound antibody was eluted with 10 mL 100 mM glycine (pH 2.5). The elution fractions were neutralized with a 2 M solution of unbuffered Tris and analyzed by Bradford assay (BioRad) to determine the fractions with the eluted antibody.

### Cytotoxicity Assay

A549 lung epithelial cells were seeded in 24-well plates (1 mL of cell suspension/well with 8×10^∧^4 A549 cells in RPMI1640 with 10% FBS). *P. aeruginosa* isolates were grown overnight in LB-MC. The next day, the A549 cells were washed 1x with PBS and 1 mL of fresh RPMI1640 with 10% FBS was added to each well. *P. aeruginosa* overnight cultures were normalized for OD_600_, diluted in PBS and used to infect the A549 cells at an MOI of 10. The infection was allowed to proceed for four hours, before the supernatant was removed and the cells were fixed with 3.7% formaldehyde in PBS (20′ at room temperature). Images of individual wells were taken using a Nikon Eclipse TE200 inverted scope. Rounded, intoxicated cells and flat cells were counted. Cytotoxicity was expressed as %rounded A549 cells. The experiment was repeated on three separate days and the results from these three assays were averaged.

### Corneal Infection Model

*P. aeruginosa* strains were grown overnight in tryptic soy broth (TSB, Becton Dickinson) and on the day of the infection back-diluted 1∶200 in tryptic soy broth and grown to an OD_600_ of 0.2. At this point 1 mL of cells was removed from the culture tube, the bacteria were pelleted by centrifugation, washed twice with PBS-MC (Dulbecco’s phosphate buffered saline (Invitrogen) with 5 mM MgCl_2_ and 0.5 mM CaCl_2_ added) and resuspended in PBS-MC. The optical density of the washed cells was determined and adjusted to correspond to a concentration of 8×10^∧^7 colony forming units (CFU)/mL. 5-week-old female C57BL/6 mice were anesthetized by i.p. injection of 0.4 mL 2,2,2-tribromoethanol (1.2%) (8 mice/bacterial strain). The cornea of one eye of the anesthetized mice was scored with three parallel, 1 mm scratches using a 26-gauge needle and 2.5 µL of the bacterial inoculum (2×10^∧^5 CFU) was spotted onto the scratched cornea. After two hours, two of the mice were sacrificed and their eyes removed. The eyes were homogenized in 1 mL of PBS by sonication in the presence of metal beads and viable bacterial CFU were determined by dilution plating. Images of infected corneas were taken one and two days post infection using a Leica dissection scope. Two days after infection, the remaining mice were euthanized, the infected eye was removed and homogenized by sonication in the presence of beads, as above. Surviving bacterial CFU were determined by dilution plating. C57BL/6 mice were purchased from The Jackson Laboratory (Bar Harbor, ME).

## Results

Previous reports had noted that not all clinical isolates of *P. aeruginosa* express the type III secretion system (T3SS), which is important for virulence, both in animal models of infection and clinical disease. In order to study the virulence of these T3SS negative strains, we screened 81 clinical isolates from the microbiology laboratory in the Pathology department at University Hospitals Case Medial Center for secretion of T3SS-related proteins. We examined production of the main T3SS effectors, ExoS, ExoT and ExoU, as well as the translocator proteins PopB and PopD, which are required for delivery of effectors into targeted host proteins. We excluded isolates from cystic fibrosis (CF) patients, since many of these strains lose expression of the type III secretion system genes during the prolonged colonization of CF patients [34]. Virulence of CF isolates is thought to depend on the formation of biofilms that are resistant to antibiotic treatment and clearance by infiltrating neutrophils [35].

**Figure 1:**
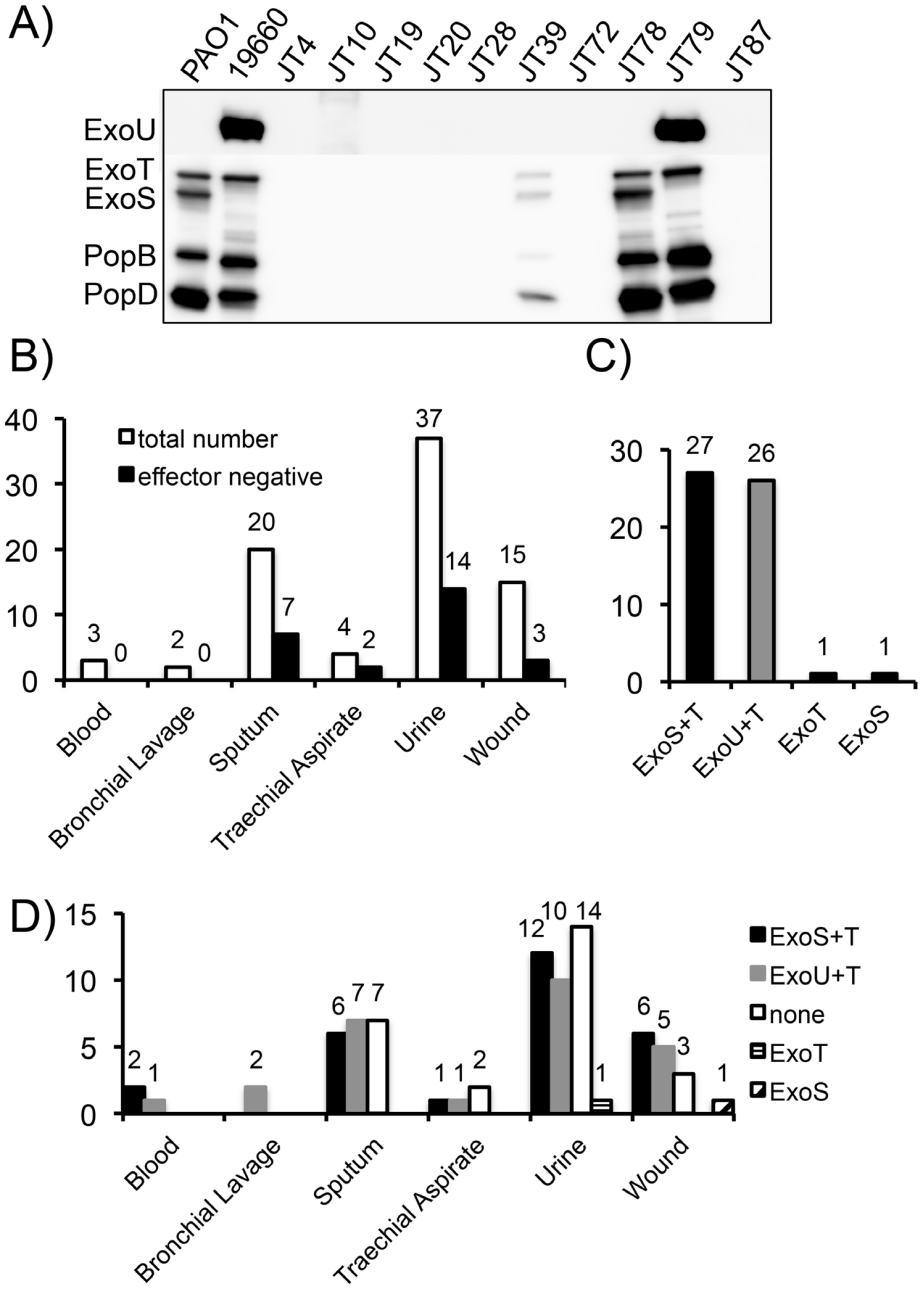
Effector expression by clinical *P. aeruginosa* isolates. A) A representative western blot showing effector (ExoS, ExoT and ExoU) as well as translocator (PopB and PopD) production in clinical isolates of *P. aeruginosa*. Protein supernatants were isolated from the indicated *P. aeruginosa* strain under T3SS inducing conditions (low calcium) and concentrated by precipitation with trichloroacetic acid before separating the proteins by SDS-PAGE and probing for the presence of the indicated effector and translocator proteins by western blot. B) Distribution of T3SS effector-positive and negative bacteria by site of isolation. C) Total distribution of produced effectors by T3SS effector-positive strains. D) Distribution of effectors produced by site from which the strains were isolated.

Clinical isolates of *P. aeruginosa* were grown *in vitro* under T3SS inducing conditions (low calcium media). Culture supernatants were then concentrated, separated by SDS-PAGE, and probed by western blot for the presence of the effectors ExoS, ExoT and ExoU, as well as the translocator proteins PopB and PopD. A representative blot is shown in Fig. 1A. Strain PAO1, which produces ExoS and ExoT, and strain 19660, which produces ExoT and ExoU, were included as controls for the western blot analysis on every experiment. Supernatant samples separated by SDS-PAGE were also stained for total protein using the fluorescent dye Sypro Ruby to ensure that the supernatants did contain secreted proteins (unpublished result). We analyzed 81 clinical isolates, which were isolated from blood, the respiratory tract, urine, or wound infections (Fig. 1B). Among the T3SS+ isolates, there were 27 secreting ExoS and ExoT, and 26 isolates secreting ExoT and ExoU. There were also two isolates that produced either ExoS only or ExoT only (Fig. 1C). The strain producing only ExoS still harbored a copy of the *exoT* gene on its chromosome as indicated by PCR, but the protein was not produced. The strain producing only ExoT, on the other hand, was negative for *exoS* and *exoU* by PCR. The distribution of effectors did not correlate with any specific site of isolation, aside from the two bronchoalveolar lavage isolates that were ExoU/ExoT producing strains. Overall, our data showed a greater proportion of ExoU-producing strains than had previously been reported [1], [22], although a recent analysis of bloodstream isolates also reported a high percentage of ExoU+ (49%) relative to ExoS+ (27%) bacteria [24].

We found that 26 of the clinical isolates were T3SS negative by western blot. However, all isolates except one harbored copies of at least one of the effector genes on the chromosome as assayed by PCR. A PCR assay for the strains assayed in Fig. 1A is included as Fig. S1A. Of the 25 strains with at least one chromosomal copy of an effector gene, 10 were *exoU*+/*exoT*+, 11 *exoS*+/*exoT*+, 2 were *exoU*+/*exoS*+/*exoT*+ and 2 only harbored the *exoT* gene, suggesting that there is no significant skew in effector complement relative to the T3SS effector-positive strains.

Notably, strain JT87 was negative for *exoS*, *exoT* and *exoU*, as well as the apparatus genes *pscU* and *pscL*, which are the outermost genes of the gene cluster encoding the secretion apparatus [36]. Strain JT87 produces pyocyanin and sequencing of a species-specific region of the 16S rDNA gene (nt 207–1123, [37]) confirmed that it is *P. aeruginosa*. One other strain of *P. aeruginosa* has been described in the literature that lacks the type III secretion genes, PA7 [38]. To determine if JT87 is also missing the T3SS apparatus genes, we used primers that bind in the flanking genes, PA1680 and *bglX* to amplify across the location of the T3SS apparatus genes. The analysis confirmed that JT87 is missing the T3SS genes since amplification yielded a product that is close to the expected size of 360bp, based on the PA7 genome (Fig. S1B).

**Figure 2:**
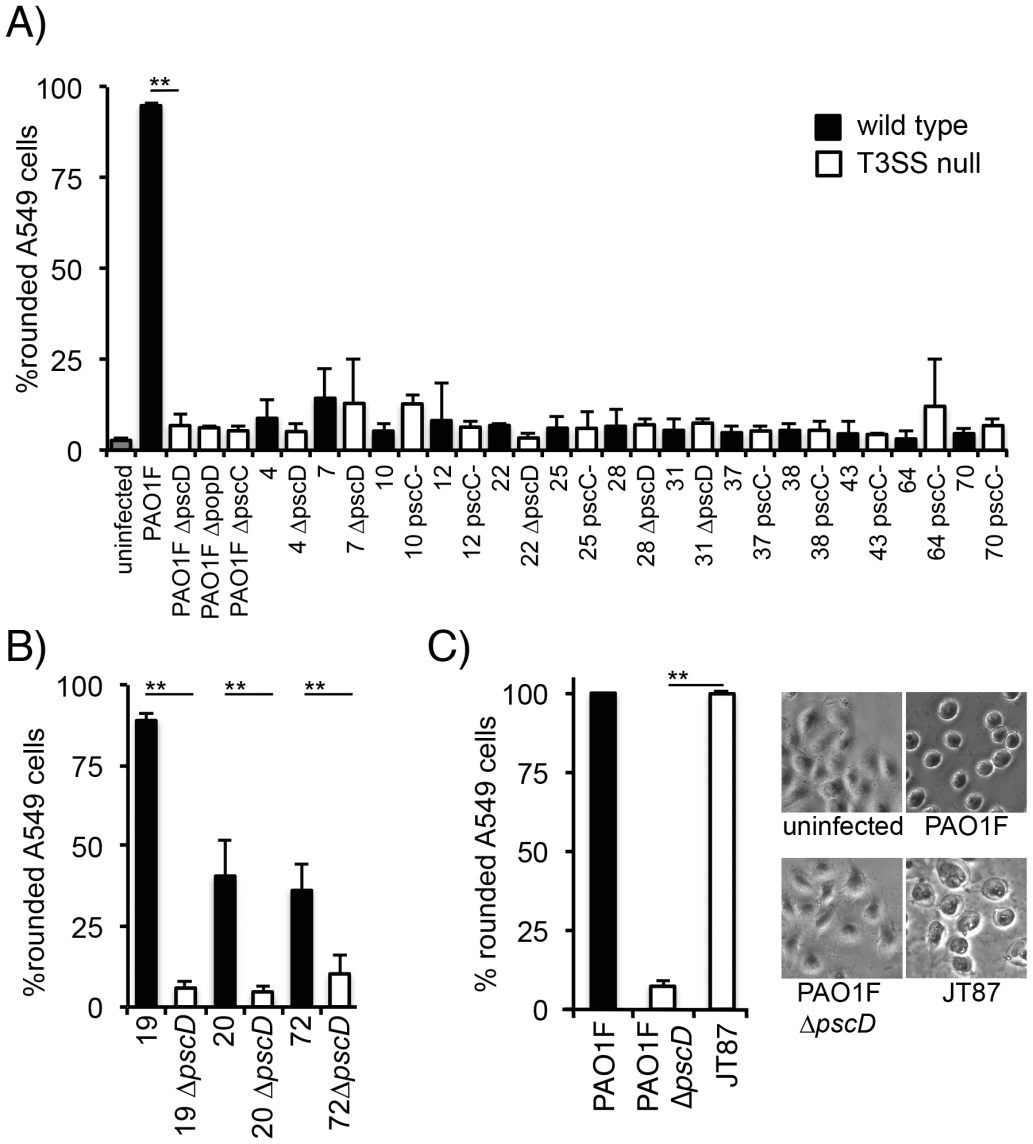
Intoxication of A549 lung epithelial cells by effector-negative clinical isolates of *P. aeruginosa*. Clinical isolates that do not secrete detectable levels of known effectors and translocators *in vitro* as well as defined T3SS null derivatives (Δ*popD*, Δ*pscD*, where the entire open reading frame was removed by an in-frame deletion, or *pscC*-, where the *pscC* open reading frame was disrupted by the insertion of a non-replicating plasmid) were tested for their ability to intoxicate A549 epithelial cells in a T3SS-dependent manner. Delivery of effector proteins was measured by assaying rounding of A549 cells by microscopic examination. Data presented is the mean of three independent experiments with standard deviation (error bar). A) Isolates with no significant cytotoxicity. B) Isolates that are cytotoxic in a T3SS-dependent manner. C) Isolate JT87 has no detectable T3SS-related genes by PCR. Representative phase contrast images of A549 cells infected with PAO1, PAO1 Δ*pscD* or JT87 are shown to the right of the graph in panel C. ** p<0.01, Student’s T-test.

The presence of effector genes in strains that do not secrete effectors *in vitro* argues that the T3SS negative phenotype stems from a regulatory phenomenon. To determine if these phenotypically T3SS negative isolates express a functional T3SS at a low level, or in a manner that is up-regulated in the presence of mammalian cells, we generated defined T3SS negative mutants and assayed their ability to intoxicate A549 cells *in vitro*. T3SS null mutations were introduced into 16 clinical isolates by deleting either the gene encoding the essential inner membrane T3SS component PscD, deleting the gene for the translocator PopD, or disrupting the gene for the essential outer membrane secretin PscC. A549 cells were then infected at an MOI of 10 for four hours and the ability to deliver effector proteins was assayed by analyzing the T3SS dependent cytopathic effect: cell rounding in ExoS producing strains or cell rounding and lysis by strains producing ExoU (Fig. 2). 13 of the 16 strains analyzed did not induce a cytopathic effect (Fig. 2A). Three isolates showed T3SS-dependent virulence (JT19, JT20, and JT72), indicating that these isolates produce a functional T3SS (Fig. 2B). Finally, isolate JT87, which lacks a T3SS, was highly cytotoxic, causing extensive cell rounding and lysis, indicated by uptake of the dye trypan blue (Fig. 2C and data not shown).

To determine if these T3SS negative clinical isolates are virulent *in vivo*, we used a well-characterized mouse model of *P. aeruginosa* corneal infection in which productive infection of the cornea depends on type III secretion [39], [40]. The corneal epithelium of C57BL/6 mice was scarified and infected with clinical isolates and their T3SS negative mutant derivatives, and virulence was defined by bacterial survival and corneal opacification related to neutrophil infiltration [41].

**Figure 3:**
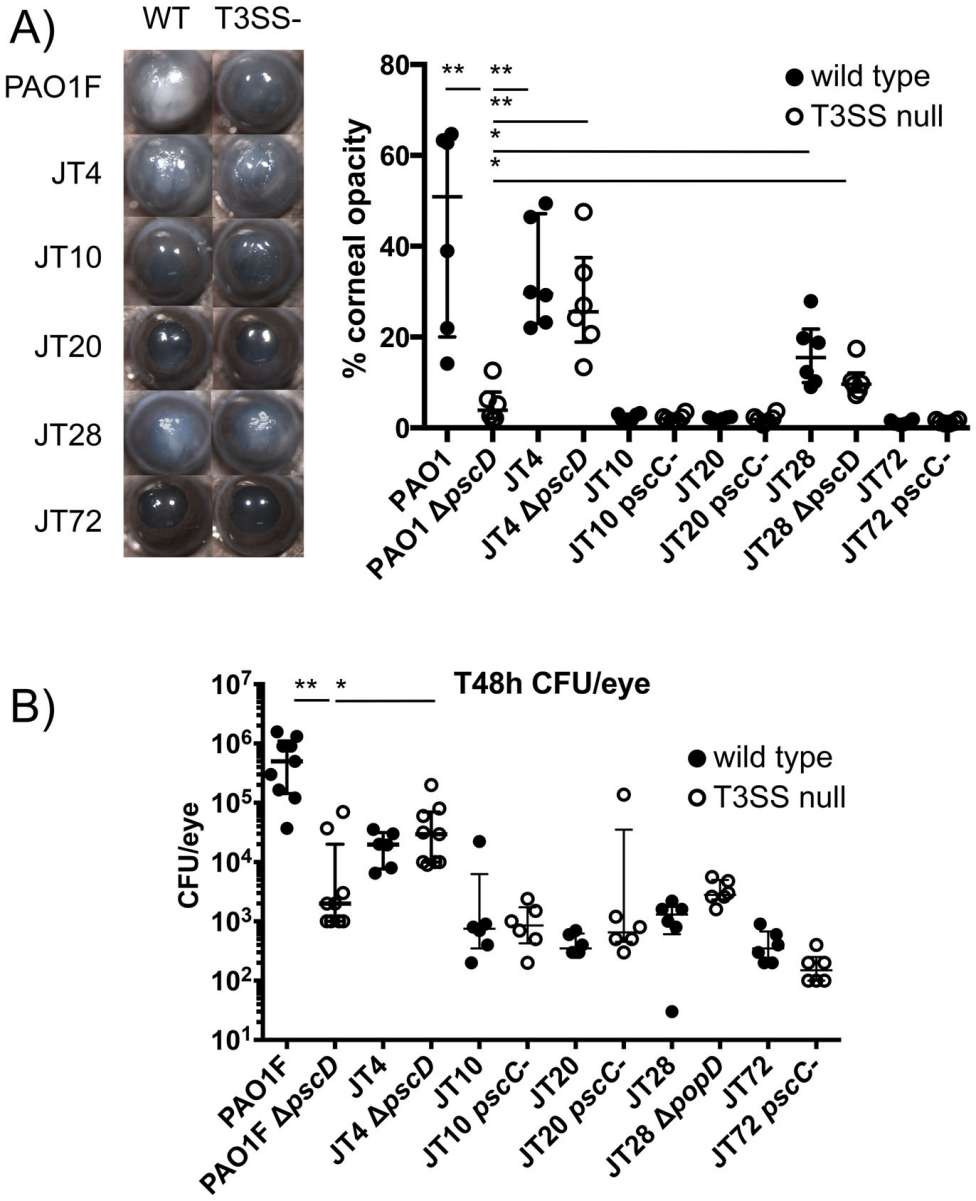
Virulence of effector-negative clinical isolates of *P. aeruginosa*. Corneas of C57BL/6 mice were scarified and infected with 2*10^∧^5 CFU/eye. A) Images of infected eyes were taken at 24 h and 48 h post-infection to assess corneal opacification due to infiltration of neutrophils. Corneal opacification was quantitated digitally using Metamorph software as described previously [11]. Opacity scores for individual eyes were plotted. The median value with interquartile range is indicated. Representative images of infected corneas are shown to the left of the graph. Statistical significance of differences was determined by Mann-Whitney test: ** p<0.01, * p<0.05 B) Mice were infected with 2*10^∧^5 CFU/eye using the scratch model of corneal infection. Bacterial load (CFU/eye) was determined 48 h after infection by euthanizing the mice, removing the infected eye, homogenizing it and plating serial dilutions on BHI agar plates. Bacterial loads for individual eyes were plotted. The median value with interquartile range is indicated. ** p<0.01, * p<0.05, Mann-Whitney test.

The animal experiments revealed a complexity of phenotypes. Infection with strains JT20 and JT72, which exhibited type III-dependent cytotoxicity in our *in vitro* assay caused very little corneal opacification and the bacteria were rapidly cleared (Fig. 3). Strain JT10, which was type III secretion negative in our *in vitro* assay was similarly cleared. Strain JT28, on the other hand, despite being cleared, induced extensive corneal opacification. In marked contrast to the other isolates, strain JT4 was able to cause disease and survive in the cornea in a T3SS-independent manner (Fig. 3). Moreover, more JT4 was recovered than our T3SS-negative control strain PAO1 Δ*pscD* (p = 0.05, Mann-Whitney test), arguing that this strain produces a T3SS-independent virulence factor(s).

## Discussion

Despite the prominent role that type III secretion plays in *P. aeruginosa* virulence in animal models, T3SS negative strains of *P. aeruginosa* are commonly isolated from the clinic. Here we analyzed 81 clinical *P. aeruginosa* isolates with respect to their production of type III secreted proteins *in vitro*. 55 of the strains expressed T3SS effectors, with an approximately equal number of strains producing ExoS and ExoT, and strains producing ExoU and ExoT. 26 of the strains produced no detectable ExoS, ExoT or ExoU, and did not produce the translocator proteins PopB and PopD. These secretion negative isolates displayed a range of virulence-related phenotypes, including type III secretion dependent and independent intoxication of epithelial cells *in *vitro, in addition to type III secretion independent disease in a corneal model of infection.

Three out of 16 of these T3SS-negative isolates were still able to deliver effectors into host cells, arguing that they encode a functional T3SS that is poorly expressed *in vitro*. Possible causes include a regulatory mutation that prevents expression of the type III secretion genes [42], a new layer of regulation that silences the T3SS genes when the bacteria are grown outside of the patient, or a mutation that renders the T3SS non-responsive to the removal of calcium from the medium. One isolate, JT87, lacked type III secretion-related genes entirely, but was highly cytotoxic *in vitro*, suggesting that this isolate produces a new toxin.

The complement of T3SS negative strains by western blot in this study was relatively high (32%) compared to previous studies, where the frequency of T3SS negative strains isolated from acute *P. aeruginosa* infections ranged from 0% [8], to 11% [2], [25], 13% [26] and 23% [23]. However, the frequency is lower than that reported in a recent survey of bacteremia isolates, 56% [24]. Loss of type III secretion is commonly associated with chronic infection. Isolates from cystic fibrosis patients tend to be T3SS negative by western blot with reported frequencies of T3SS-negative strains including 61% [2] and 72% [43]. In a recent survey of isolates from chronically infected cystic fibrosis patients, only 45% of *P. aeruginosa* isolates from first infected CF patients secreted T3SS proteins. The percentage declined to 29% in chronically infected children and 12% in chronically infected adult CF patients [34].

Analysis of effector distribution in a panel of clinical and environmental isolates found that approximately 72% of *P. aeruginosa* strains harbored *exoS* effector genes, whereas 28% harbored *exoU*. Presence of *exoS* and *exoU* was found to be mutually exclusive in almost all instances [1], [22]. Altered effector distribution has been reported for cystic fibrosis isolates and for corneal infections. Patient isolates from cystic fibrosis patients tend to favor ExoS-producing strains [1], [22]. Isolates from corneal infections tend to have more ExoU-producing strains when derived from contact-lens related infections (50–66%, [4], [44], [45]), but not when the infection occurred due to injury unrelated to contact-lens wear [8], [21]. Two studies, focusing mainly on ventilator associated pneumonia, found the distribution of ExoS-producing strains compared to ExoU-producing strains to be closer, with 59%(S+)/37%(U+) and 41%(S+)/33%(U+), respectively [2], [23]. Here we found the ratio of ExoS-producing to ExoU-producing strains to be equal. This high prevalence of ExoU-producing strains may be a reflection of our focus on isolates from acute infections. ExoU-producing strains have been linked to more severe disease [46].

Three strains we analyzed, JT4, JT28 and JT87, showed novel phenotypes. Infection with JT28 resulted in significant corneal opacification, despite clearance of the infection. Since opacification is primarily related to infiltration of neutrophils, not bacterial replication [41], this would suggest that JT28 infection results in delivery of pro-inflammatory pathogen-associated molecular pattern molecules (PAMPs) that are either more potent than those delivered by other *P. aeruginosa* isolates studied here, or that linger once clearance of the infecting bacteria has begun, resulting in continued pro-inflammatory signaling. JT4, on the other hand, persisted in the cornea and caused disease in a T3SS-independent manner, arguing that this strain either expresses a novel virulence factor or overexpresses a known virulence factor, such as proteases or lipases that can contribute to the virulence of *P. aeruginosa* [47]–[53]. Strain JT87 was the only clinical isolate found to be devoid of T3SS-related genes by PCR, reminiscent of the taxonomic outlier *P. aeruginosa* strain PA7, whose genome sequence was recently reported and found to be devoid of T3SS apparatus and effector genes [38]. Interestingly, JT87 was highly cytolytic *in vitro*, arguing that it encodes a novel virulence factor. Beyond promoting T3SS-independent disease, such new virulence mechanisms may also augment the virulence of type III secretion positive *P. aeruginosa* isolates. It has already been noted that accessory factors exist that modulate the virulence of T3SS-positive strains [54].

Taken together, our data demonstrate that type III secretion negative isolates of *P. aeruginosa* can not only survive *in vivo*, but also cause disease. Our discovery of a strain that can cause significant inflammation despite being cleared by the immune system highlights that treatment of some infections, particular in the eye where a significant portion of the disease can be attributed to damage caused by infiltrating neutrophils, could benefit from anti-inflammatory therapy. Moreover our data suggest that new virulence mechanisms remain to be discovered that allow *P. aeruginosa* to persist and cause disease in a type III secretion independent manner.

## Supporting Information

Figure S1 **PCR analysis of T3SS gene distribution.** A) Presence of *exoU*, *exoS*, *exoT*, *pscL* and *pscU* were probed by colony PCR. B) Presence of the genes encoding the T3SS apparatus was probed using primers that bind in the flanking genes, PA1689 and *bglX*. Wild-type PAO1F and a mutant derivative in which the T3SS apparatus genes had been deleted (Δ*pscUL*) were used as controls. Presence of *pscL* and *pscU* in these strains was probed with the same ORF-specific primer set used in A). A schematic showing the organization of PA1689, *bglX* and the intervening T3SS-apparatus genes is shown below the gel.
(TIF)

## References

[1] Feltman H, Schulert G, Khan S, Jain M, Peterson L, et al (2001) Prevalence of type III secretion genes in clinical and environmental isolates of Pseudomonas aeruginosa. Microbiology 147: 2659–2669. 11577145 10.1099/00221287-147-10-2659

[2] Roy-Burman A, Savel RH, Racine S, Swanson BL, Revadigar NS, et al (2001) Type III protein secretion is associated with death in lower respiratory and systemic Pseudomonas aeruginosa infections. J Infect Dis 183: 1767–1774. 11372029 10.1086/320737

[3] Wong-Beringer A, Wiener-Kronish J, Lynch S, Flanagan J (2008) Comparison of type III secretion system virulence among fluoroquinolone-susceptible and -resistant clinical isolates of Pseudomonas aeruginosa. Clinical microbiology and infection : the official publication of the European Society of Clinical Microbiology and Infectious Diseases 14: 330–336. 10.1111/j.1469-0691.2007.01939.x 18190571

[4] Cowell BA, Weissman BA, Yeung KK, Johnson L, Ho S, et al (2003) Phenotype of Pseudomonas aeruginosa isolates causing corneal infection between 1997 and 2000. Cornea 22: 131–134. 12605047 10.1097/00003226-200303000-00010

[5] Cheng KH, Leung SL, Hoekman HW, Beekhuis WH, Mulder PG, et al (1999) Incidence of contact-lens-associated microbial keratitis and its related morbidity. Lancet 354: 181–185. 10421298 10.1016/S0140-6736(98)09385-4

[6] Stapleton F, Keay L, Jalbert I, Cole N (2007) The epidemiology of contact lens related infiltrates. Optom Vis Sci 84: 257–272. 17435509 10.1097/OPX.0b013e3180485d5f

[7] Willcox MD (2007) Pseudomonas aeruginosa infection and inflammation during contact lens wear: a review. Optom Vis Sci 84: 273–278. 17435510 10.1097/OPX.0b013e3180439c3e

[8] Karthikeyan RS, Priya JL, Leal SM Jr, Toska J, Rietsch A, et al (2013) Host response and bacterial virulence factor expression in Pseudomonas aeruginosa and Streptococcus pneumoniae corneal ulcers. PloS one 8: e64867. 23750216 10.1371/journal.pone.0064867 PMC3672173

[9] Hauser AR (2009) The type III secretion system of Pseudomonas aeruginosa: infection by injection. Nat Rev Microbiol 7: 654–665. 19680249 10.1038/nrmicro2199 PMC2766515

[10] Diaz MH, Hauser AR (2010) Pseudomonas aeruginosa cytotoxin ExoU is injected into phagocytic cells during acute pneumonia. Infection and immunity 78: 1447–1456. 20100855 10.1128/IAI.01134-09 PMC2849415

[11] Sun Y, Karmakar M, Taylor PR, Rietsch A, Pearlman E (2012) ExoS and ExoT ADP ribosyltransferase activities mediate Pseudomonas aeruginosa keratitis by promoting neutrophil apoptosis and bacterial survival. Journal of immunology 188: 1884–1895. 10.4049/jimmunol.1102148 PMC3273577 22250085

[12] Barbieri JT, Sun J (2004) Pseudomonas aeruginosa ExoS and ExoT. Rev Physiol Biochem Pharmacol 152: 79–92. 15375697 10.1007/s10254-004-0031-7

[13] Sato H, Frank DW, Hillard CJ, Feix JB, Pankhaniya RR, et al (2003) The mechanism of action of the Pseudomonas aeruginosa-encoded type III cytotoxin, ExoU. Embo J 22: 2959–2969. 12805211 10.1093/emboj/cdg290 PMC162142

[14] Yahr TL, Vallis AJ, Hancock MK, Barbieri JT, Frank DW (1998) ExoY, an adenylate cyclase secreted by the Pseudomonas aeruginosa type III system. Proc Natl Acad Sci U S A 95: 13899–13904. 9811898 10.1073/pnas.95.23.13899 PMC24955

[15] Hauser AR, Kang PJ, Engel JN (1998) PepA, a secreted protein of Pseudomonas aeruginosa, is necessary for cytotoxicity and virulence. Mol Microbiol 27: 807–818. 9515706 10.1046/j.1365-2958.1998.00727.x

[16] Kurahashi K, Kajikawa O, Sawa T, Ohara M, Gropper MA, et al (1999) Pathogenesis of septic shock in Pseudomonas aeruginosa pneumonia. J Clin Invest 104: 743–750. 10491409 10.1172/JCI7124 PMC408437

[17] Sawa T, Yahr TL, Ohara M, Kurahashi K, Gropper MA, et al (1999) Active and passive immunization with the Pseudomonas V antigen protects against type III intoxication and lung injury. Nat Med 5: 392–398. 10202927 10.1038/7391

[18] Garrity-Ryan L, Kazmierczak B, Kowal R, Comolli J, Hauser A, et al (2000) The arginine finger domain of ExoT contributes to actin cytoskeleton disruption and inhibition of internalization of Pseudomonas aeruginosa by epithelial cells and macrophages. Infect Immun 68: 7100–7113. 11083836 10.1128/iai.68.12.7100-7113.2000 PMC97821

[19] Lee VT, Smith RS, Tummler B, Lory S (2005) Activities of Pseudomonas aeruginosa effectors secreted by the Type III secretion system in vitro and during infection. Infect Immun 73: 1695–1705. 15731070 10.1128/IAI.73.3.1695-1705.2005 PMC1064929

[20] Vance RE, Rietsch A, Mekalanos JJ (2005) Role of the type III secreted exoenzymes S, T, and Y in systemic spread of Pseudomonas aeruginosa PAO1 in vivo. Infect Immun 73: 1706–1713. 15731071 10.1128/IAI.73.3.1706-1713.2005 PMC1064930

[21] Choy MH, Stapleton F, Willcox MD, Zhu H (2008) Comparison of virulence factors in Pseudomonas aeruginosa strains isolated from contact lens- and non-contact lens-related keratitis. Journal of medical microbiology 57: 1539–1546. 19018027 10.1099/jmm.0.2008/003723-0

[22] Bradbury RS, Roddam LF, Merritt A, Reid DW, Champion AC (2010) Virulence gene distribution in clinical, nosocomial and environmental isolates of Pseudomonas aeruginosa. Journal of medical microbiology 59: 881–890. 20430902 10.1099/jmm.0.018283-0

[23] Hauser AR, Cobb E, Bodi M, Mariscal D, Valles J, et al (2002) Type III protein secretion is associated with poor clinical outcomes in patients with ventilator-associated pneumonia caused by Pseudomonas aeruginosa. Crit Care Med 30: 521–528. 11990909 10.1097/00003246-200203000-00005

[24] El-Solh AA, Hattemer A, Hauser AR, Alhajhusain A, Vora H (2012) Clinical outcomes of type III Pseudomonas aeruginosa bacteremia. Crit Care Med 40: 1157–1163. 22080633 10.1097/CCM.0b013e3182377906 PMC3288436

[25] Wareham DW, Curtis MA (2007) A genotypic and phenotypic comparison of type III secretion profiles of Pseudomonas aeruginosa cystic fibrosis and bacteremia isolates. International journal of medical microbiology : IJMM 297: 227–234. 17412636 10.1016/j.ijmm.2007.02.004

[26] Zhuo H, Yang K, Lynch SV, Dotson RH, Glidden DV, et al (2008) Increased mortality of ventilated patients with endotracheal Pseudomonas aeruginosa without clinical signs of infection. Critical care medicine 36: 2495–2503. 18679122 10.1097/CCM.0b013e318183f3f8

[27] Miller VL, Mekalanos JJ (1988) A novel suicide vector and its use in construction of insertion mutations: osmoregulation of outer membrane proteins and virulence determinants in Vibrio cholerae requires toxR. J Bacteriol 170: 2575–2583. 2836362 10.1128/jb.170.6.2575-2583.1988 PMC211174

[28] Bleves S, Soscia C, Nogueira-Orlandi P, Lazdunski A, Filloux A (2005) Quorum sensing negatively controls type III secretion regulon expression in Pseudomonas aeruginosa PAO1. J Bacteriol 187: 3898–3902. 15901720 10.1128/JB.187.11.3898-3902.2005 PMC1112058

[29] Hobden JA, Gupta SK, Masinick SA, Wu X, Kernacki KA, et al (1996) Anti-receptor antibodies inhibit Pseudomonas aeruginosa binding to the cornea and prevent corneal perforation. Immunol Cell Biol 74: 258–264. 8799726 10.1038/icb.1996.46

[30] Cisz M, Lee PC, Rietsch A (2008) ExoS controls the cell contact-mediated switch to effector secretion in Pseudomonas aeruginosa. J Bacteriol 190: 2726–2738. 18039770 10.1128/JB.01553-07 PMC2293250

[31] Rietsch A, Vallet-Gely I, Dove SL, Mekalanos JJ (2005) ExsE, a secreted regulator of type III secretion genes in Pseudomonas aeruginosa. Proc Natl Acad Sci U S A 102: 8006–8011. 15911752 10.1073/pnas.0503005102 PMC1142391

[32] Warrens AN, Jones MD, Lechler RI (1997) Splicing by overlap extension by PCR using asymmetric amplification: an improved technique for the generation of hybrid proteins of immunological interest. Gene 186: 29–35. 9047341 10.1016/s0378-1119(96)00674-9

[33] Campo N, Rudner DZ (2006) A branched pathway governing the activation of a developmental transcription factor by regulated intramembrane proteolysis. Mol Cell 23: 25–35. 16818230 10.1016/j.molcel.2006.05.019

[34] Jain M, Bar-Meir M, McColley S, Cullina J, Potter E, et al (2008) Evolution of Pseudomonas aeruginosa type III secretion in cystic fibrosis: a paradigm of chronic infection. Transl Res 152: 257–264. 19059160 10.1016/j.trsl.2008.10.003 PMC2628760

[35] Hoiby N, Ciofu O, Bjarnsholt T (2010) Pseudomonas aeruginosa biofilms in cystic fibrosis. Future Microbiol 5: 1663–1674. 21133688 10.2217/fmb.10.125

[36] Stover CK, Pham XQ, Erwin AL, Mizoguchi SD, Warrener P, et al (2000) Complete genome sequence of Pseudomonas aeruginosa PA01, an opportunistic pathogen. Nature 406: 959–964. 10984043 10.1038/35023079

[37] Spilker T, Coenye T, Vandamme P, LiPuma JJ (2004) PCR-based assay for differentiation of Pseudomonas aeruginosa from other Pseudomonas species recovered from cystic fibrosis patients. J Clin Microbiol 42: 2074–2079. 15131172 10.1128/JCM.42.5.2074-2079.2004 PMC404678

[38] Roy PH, Tetu SG, Larouche A, Elbourne L, Tremblay S, et al (2010) Complete genome sequence of the multiresistant taxonomic outlier Pseudomonas aeruginosa PA7. PloS one 5: e8842. 20107499 10.1371/journal.pone.0008842 PMC2809737

[39] Lee EJ, Cowell BA, Evans DJ, Fleiszig SM (2003) Contribution of ExsA-regulated factors to corneal infection by cytotoxic and invasive Pseudomonas aeruginosa in a murine scarification model. Invest Ophthalmol Vis Sci 44: 3892–3898. 12939306 10.1167/iovs.02-1302

[40] Lee EJ, Evans DJ, Fleiszig SM (2003) Role of Pseudomonas aeruginosa ExsA in penetration through corneal epithelium in a novel in vivo model. Invest Ophthalmol Vis Sci 44: 5220–5227. 14638720 10.1167/iovs.03-0229

[41] Sun Y, Karmakar M, Roy S, Ramadan RT, Williams SR, et al (2010) TLR4 and TLR5 on Corneal Macrophages Regulate Pseudomonas aeruginosa Keratitis by Signaling through MyD88-Dependent and -Independent Pathways. J Immunol 185: 4272–4283. 20826748 10.4049/jimmunol.1000874 PMC3392180

[42] Yahr TL, Wolfgang MC (2006) Transcriptional regulation of the Pseudomonas aeruginosa type III secretion system. Mol Microbiol 62: 631–640. 16995895 10.1111/j.1365-2958.2006.05412.x

[43] Dacheux D, Toussaint B, Richard M, Brochier G, Croize J, et al (2000) Pseudomonas aeruginosa cystic fibrosis isolates induce rapid, type III secretion-dependent, but ExoU-independent, oncosis of macrophages and polymorphonuclear neutrophils. Infect Immun 68: 2916–2924. 10768989 10.1128/iai.68.5.2916-2924.2000 PMC97504

[44] Lomholt JA, Poulsen K, Kilian M (2001) Epidemic population structure of Pseudomonas aeruginosa: evidence for a clone that is pathogenic to the eye and that has a distinct combination of virulence factors. Infection and immunity 69: 6284–6295. 11553572 10.1128/IAI.69.10.6284-6295.2001 PMC98763

[45] Winstanley C, Kaye SB, Neal TJ, Chilton HJ, Miksch S, et al (2005) Genotypic and phenotypic characteristics of Pseudomonas aeruginosa isolates associated with ulcerative keratitis. Journal of medical microbiology 54: 519–526. 15888458 10.1099/jmm.0.46005-0

[46] Schulert GS, Feltman H, Rabin SD, Martin CG, Battle SE, et al (2003) Secretion of the toxin ExoU is a marker for highly virulent Pseudomonas aeruginosa isolates obtained from patients with hospital-acquired pneumonia. The Journal of infectious diseases 188: 1695–1706. 14639541 10.1086/379372

[47] Vasil ML, Graham LM, Ostroff RM, Shortridge VD, Vasil AI (1991) Phospholipase C: molecular biology and contribution to the pathogenesis of Pseudomonas aeruginosa. Antibiot Chemother 44: 34–47. 1801644 10.1159/000420295

[48] Twining SS, Kirschner SE, Mahnke LA, Frank DW (1993) Effect of Pseudomonas aeruginosa elastase, alkaline protease, and exotoxin A on corneal proteinases and proteins. Invest Ophthalmol Vis Sci 34: 2699–2712. 8344792

[49] Preston MJ, Seed PC, Toder DS, Iglewski BH, Ohman DE, et al (1997) Contribution of proteases and LasR to the virulence of Pseudomonas aeruginosa during corneal infections. Infect Immun 65: 3086–3090. 9234758 10.1128/iai.65.8.3086-3090.1997 PMC175435

[50] Cowell BA, Twining SS, Hobden JA, Kwong MS, Fleiszig SM (2003) Mutation of lasA and lasB reduces Pseudomonas aeruginosa invasion of epithelial cells. Microbiology 149: 2291–2299. 12904569 10.1099/mic.0.26280-0

[51] Alarcon I, Kwan L, Yu C, Evans DJ, Fleiszig SM (2009) Role of the corneal epithelial basement membrane in ocular defense against Pseudomonas aeruginosa. Infect Immun 77: 3264–3271. 19506010 10.1128/IAI.00111-09 PMC2715680

[52] Engel LS, Hill JM, Moreau JM, Green LC, Hobden JA, et al (1998) Pseudomonas aeruginosa protease IV produces corneal damage and contributes to bacterial virulence. Invest Ophthalmol Vis Sci 39: 662–665. 9501882

[53] Tang A, Caballero AR, Marquart ME, O’Callaghan RJ (2013) Pseudomonas aeruginosa small protease (PASP), a keratitis virulence factor. Invest Ophthalmol Vis Sci 54: 2821–2828. 23548618 10.1167/iovs.13-11788 PMC3632270

[54] Battle SE, Meyer F, Rello J, Kung VL, Hauser AR (2008) Hybrid pathogenicity island PAGI-5 contributes to the highly virulent phenotype of a Pseudomonas aeruginosa isolate in mammals. Journal of bacteriology 190: 7130–7140. 18757543 10.1128/JB.00785-08 PMC2580712

