# Supplementary figures and images for: Diversity of Virulence Phenotypes among Type III Secretion Negative Pseudomonas aeruginosa Clinical Isolates
Source: PLoS One. 2014 Jan 23;9(1):e86829. doi: 10.1371/journal.pone.0086829 (PMC3900666; doi:10.1371/journal.pone.0086829)

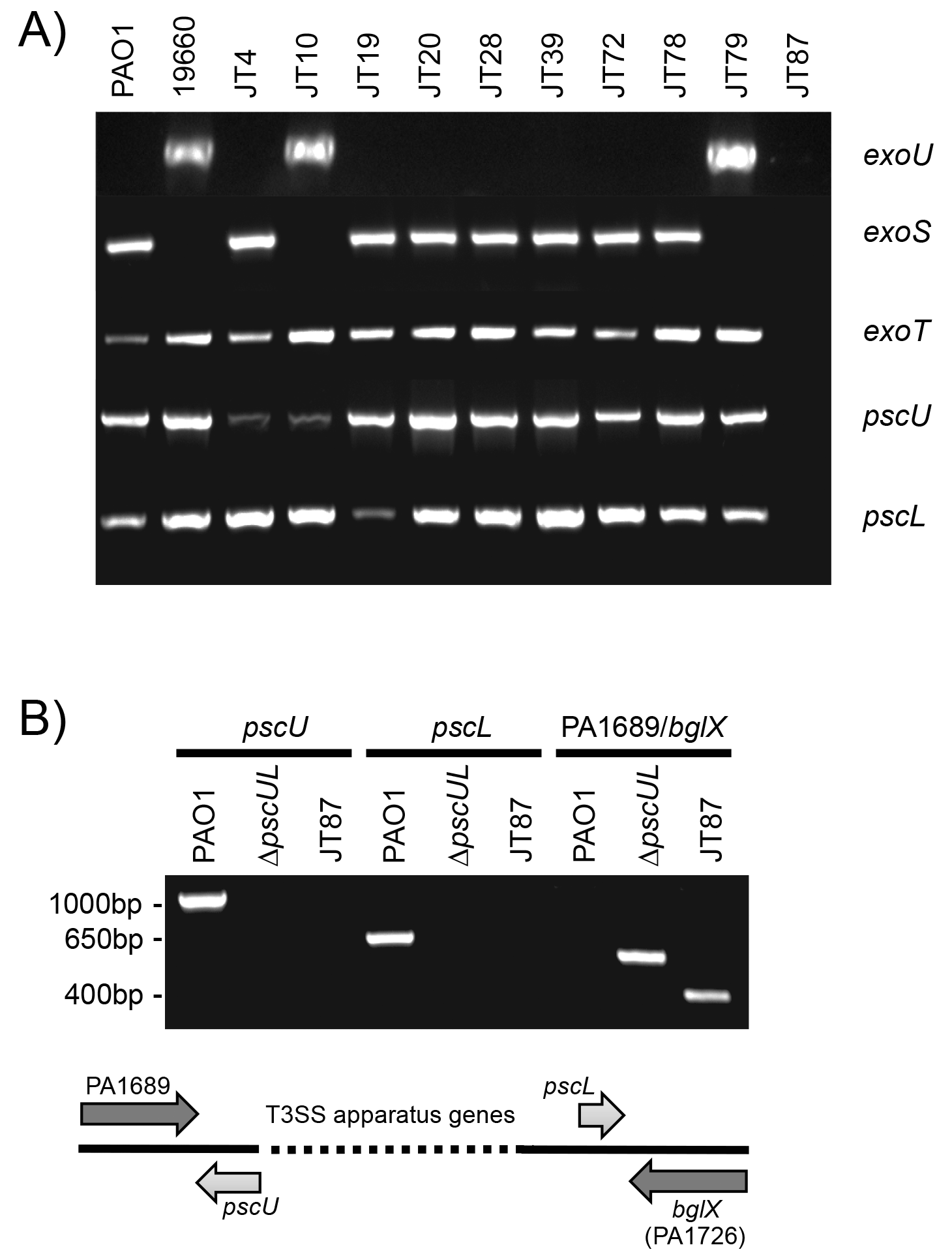

Supplement: Figure S1 — PCR analysis of T3SS gene distribution. A) Presence of exoU, exoS, exoT, pscL and pscU were probed by colony PCR. B) Presence of the genes encoding the T3SS apparatus was probed using primers that bind in the flanking genes, PA1689 and bglX. Wild-type PAO1F and a mutant derivative in which the T3SS apparatus genes had been deleted (ΔpscUL) were used as controls. Presence of pscL and pscU in these strains was probed with the same ORF-specific primer set used in A). A schematic showing the organization of PA1689, bglX and the intervening T3SS-apparatus genes is shown below the gel. (TIF) [file pone.0086829.s001.tif]
